# Synopsis of fruit-piercing moths of the genus *Eudocima* (Lepidoptera, Erebidae) from Colombia

**DOI:** 10.3897/zookeys.953.50709

**Published:** 2020-07-27

**Authors:** Sergio Vargas-Fonseca, Yenny Correa-Carmona, José Mauricio Montes-Rodríguez, Humberto Calero-Mejía, Alberto Zilli

**Affiliations:** 1 Laboratorio de Entomología, Departamento de Biología, Pontificia Universidad Javeriana, Carrera 7, No. 43–82, Bogotá, Colombia Pontificia Universidad Javeriana Bogota Colombia; 2 Grupo de Entomología Universidad de Antioquia (GEUA), Apartado Aéreo 1226 Medellín, Colombia Grupo de Entomología Universidad de Antioquia Medellin Colombia; 3 Corporación Colombiana de Investigación Agropecuaria - AGROSAVIA. Centro de Investigación La Suiza – Km 32 vía al mar, vereda Galápagos, Rionegro, Santander, Colombia Corporación Colombiana de Investigación Agropecuaria Bucaramanga Colombia; 4 Grupo de Investigación en Ecología y Conservación Neotropical, Fundación de apoyo educativo e investigativo SAMANEA, Cali, Colombia Fundación de apoyo educativo e investigativo SAMANEA Cali Colombia; 5 Natural History Museum, Life Sciences, DC2-2N, Cromwell Road, SW7 5BD, London, UK Natural History Museum London United Kingdom

**Keywords:** Biodiversity, distribution, El Niño-Southern Oscillation (ENSO), entomological collections, fruit pest, taxonomy

## Abstract

In order to provide information about the diversity and distribution of *Eudocima* species in Colombia, 261 specimens deposited in entomological collections were examined and identified. We found seven of the eight species of *Eudocima* recorded in the Neotropics: *E. anguina*, *E. colubra*, *E. collusoria*, *E. memorans* and *E. serpentifera*, all being recorded for the first time from the country. We provide a list of the species, comments on the biology and distribution data, illustrations of the adults, and keys for species identification.

## Introduction

The fruit-piercing moth genus *Eudocima* Billberg, 1820 (Erebidae, Calpinae) encompasses approximately 50 species distributed throughout tropical and subtropical regions of the world (Zaspel and Branham 2008, Zilli et al. 2017), with eight species occurring in the Neotropics (Zilli and Hogenes 2002). They are generally large-sized and with variably colored patterns, mainly cryptic on the forewings and with bright yellow-orange hindwings, and at least in the Neotropics the species always bear dark spots or bands on the hindwings. Historically, neotropical species of *Eudocima* have been placed in several genera according to differences in their habitus, e.g., *Elygea* Billberg, 1820, *Othreis* Hübner, [1823], *Trissophaes* Hübner, [1823] and *Ophideres* Boisduval, 1832, all now subsumed under *Eudocima*. Like other genera of the subfamily Calpinae, they possess sclerotized and apically sharpened proboscis with tearing hooks, with which they pierce fruits to feed on their juices. Accordingly, unlike other groups of agriculturally important Lepidoptera, it is the adults that damage crops, which in this case takes place due to rotting agents such as fungi and bacteria that penetrate the holes that they leave onto the fruit skin.

In Asian countries and islands of the Pacific, fruit-piercing *Eudocima* are frequently reported as damaging crops, while in the Americas they are only sporadically mentioned as pests (Hernández-Ruiz et al. 2017, Montes et al. 2018), and information about this group is generally scarce.

In Colombia, *E. apta* (Walker, [1858]) and *E. procus* (Cramer, 1777), two species widely distributed in the Neotropics, were recently reported as occasional citrus pests (Montes et al. 2018), but the diversity and distribution of this genus in the country are unknown. Vouchers in biological collections can provide important information about the spatial and temporal distribution of species. In Colombia, many universities and research centers are maintaining biological collections where specimens from monitoring programs and ecological sampling are regularly being deposited. It was therefore expected that these colorful large moths would be well represented in such collections.

The aim of this work is to report information on *Eudocima* from specimen data preserved in collections and produce a checklist and an identification key to species occurring in Colombia. Additionally, we provide information about the environmental variables determining species distribution. This information will facilitate a baseline for planning ecological studies and taking phytosanitary actions in case of the detection of pest species in fruit orchards. Furthermore, the checklist could assist with the resolution of environmental factors determining presence of these moths in cultivations and enable the development of models to forecast their occurrence in agroecosystems.

## Material and methods

The checklist presented here collates literature records for Colombia based on Walker ([1858]) and Montes et al. (2018) with specimen data drawn from the following entomological collections:

**CEUA** Colección de Entomología de la Universidad de Antioquia, Medellín, Colombia

**CTNI** Colección Taxonómica Nacional de Insectos “Luis María Murillo” – Agrosavia, Mosquera, Colombia

**HNSA** Haus der Natur, Salzburg, Austria

**ICN-MHN** Instituto de Ciencias Naturales-Colección de Zoología, Universidad Nacional, Bogotá, Colombia

**JFLC** Colección Privada LeCrom, Bogotá, Colombia

**MEFLG** Museo Entomológico Francisco Luis Gallego, Universidad Nacional de Colombia, Medellín, Colombia

**MHN-UIS** Museo de Historia Natural, Universidad Industrial de Santander, Bucaramanga, Colombia

**MLS** Museo de La Salle, Bogotá, Colombia

**MPUJ_ENT** Museo Javeriano de Historia Natural, Pontificia Universidad Javeriana, Bogotá, Colombia

**MUSENUV** Colección entomológica Universidad del Valle, Cali, Colombia

**NHMUK** Natural History Museum, London, UK (historically BMNH, British Museum Natural History)

**UNAB** Museo Entomológico Facultad de Agronomía, Universidad Nacional de Colombia, Bogotá, Colombia

For taxonomic identification the original descriptions and the checklist of Zilli and Hogenes (2002) were used as a first guide, subject to comparisons with materials in NHMUK. The generic classification follows Zaspel and Branham (2008).

Relevant information was retrieved wherever possible from specimen labels in order to arrange distribution maps with occurrence data and assess biological and ecological traits of species. The occurrence maps were constructed using ArcMap 10.2 (Esri) and a digital elevation model of the Shuttle Radar Topography Mission, which has a resolution of 250 m (Jarvis et al. 2008).

Specimens were photographed in both dorsal and ventral views with a Camera Canon SX50 HS. The photographs were edited using Adobe Photoshop version 20.0.

## Results

**Figure 1.**
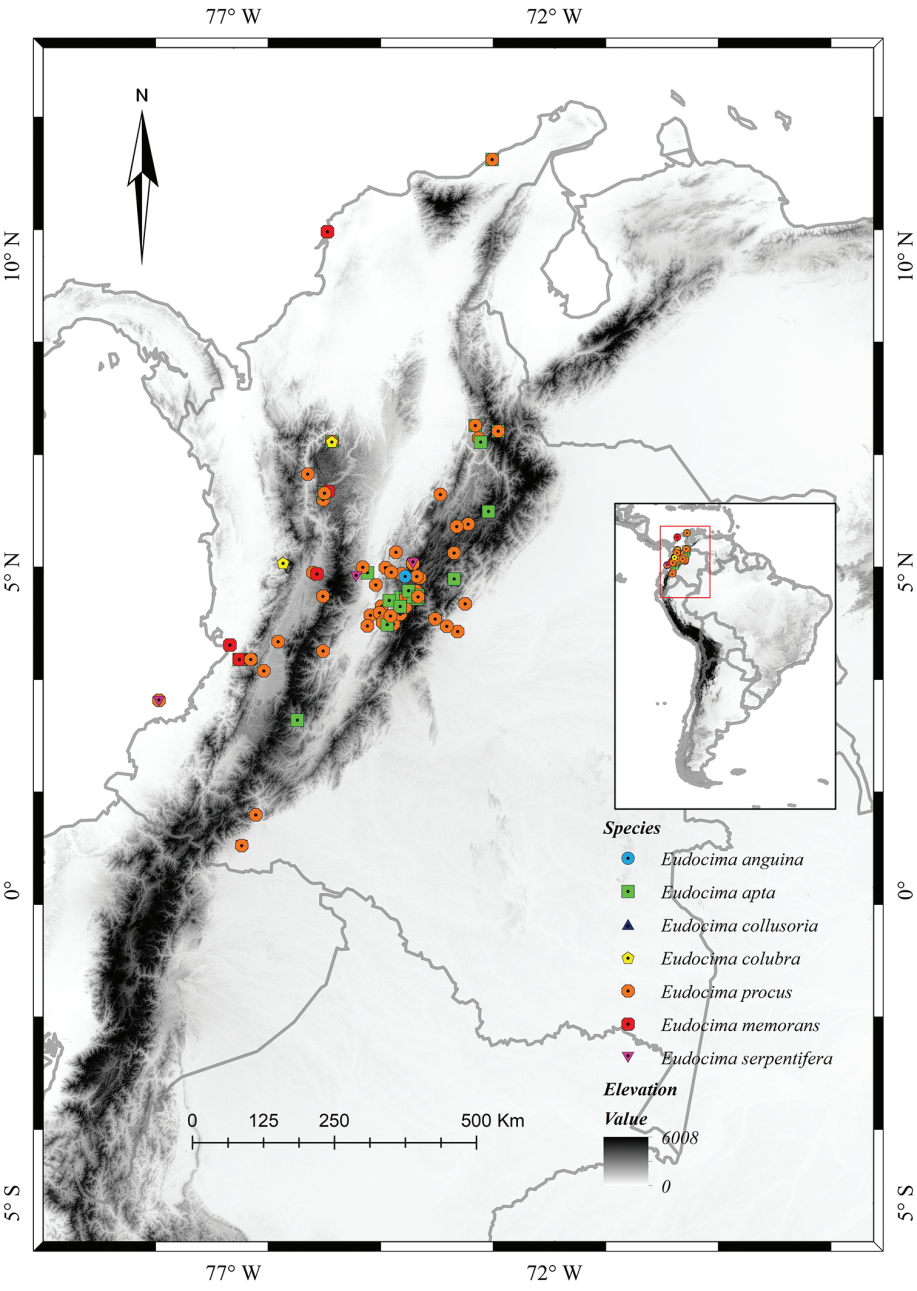
Distribution of *Eudocima* fruit-piercing moths in Colombia.

A total of 261 fruit-piercing moths of the genus *Eudocima* were studied in this work. They represent seven species. Of these, *E. anguina* (Schaus, 1911), *E. collusoria* (Cramer, 1777), *E. colubra* (Schaus, 1911), *E. memorans* (Walker, [1858]) and *E. serpentifera* (Walker, [1858]) are recorded for the first time from Colombia. The records originate from 15 departments, mostly from the Andean region of the country (Fig. 1).

### Key to species of *Eudocima* recorded in Colombia

**Table:** 

1	Hindwing without marginal band and with two sinuous bands beyond basal dark area	***E. procus***
–	Hindwing with a black marginal band	**2**
2	Hindwing with a discal circular spot	***E. apta***
–	Hindwing with a discal band	**3**
3	Discal band of hindwing straight and short	***E. anguina***
–	Discal band of hindwing lobed into an “m”-shape	**4**
4	Discal band of hindwing ending well before wing margin	***E. serpentifera***
–	Discal band of hindwing reaching wing margin	**5**
5	Discal band of hindwing with inner margin nearly straight	***E. collusoria***
–	Discal band of hindwing with inner margin distinctly concave	**6**
6	Forewing crossed by pale transverse lines, postmedial split into more waves before slightly rounded apex	***E. memorans***
–	Forewing crossed by dark transverse lines, postmedial distinct from fairly acute apex	***E. colubra***

### Annotated list of Colombian *Eudocima*

#### Eudocima anguina

Taxon classificationAnimaliaLepidopteraNoctuidae

(Schaus, 1911) (Trissophaes)

C9FBF8F2-7C2E-5080-9B96-BD186E41D907

Fig. 2A, B

##### Material examined.

Colombia. **Cundinamarca**: 1♂; San Francisco; vda. Arrayán, Finca Buena vista; 4.9333, -74.2833; 1520 m; 20 Jul. 2014; L. Tarazona leg.; light trap; UNAB.

##### Comments.

This species is characterized by a short discal band on the hindwings. Schaus (1911) discusses the possibility that *Eudocima anguina* and *Eudocima collusoria* are conspecific, which would make *Trissophaes anguina* Schaus, 1911 a synonym of Phalaena (Noctua) collusoria Cramer, 1777; however, Zilli and Hogenes (2002) retained both as valid species. The hostplant and life cycle are unknown.

##### Distribution.

Costa Rica (Schaus 1911) and Colombia.

##### Remarks.

This species is herewith recorded for the first time from Colombia, in the locality of San Francisco, Cundinamarca.

**Figure 2.**
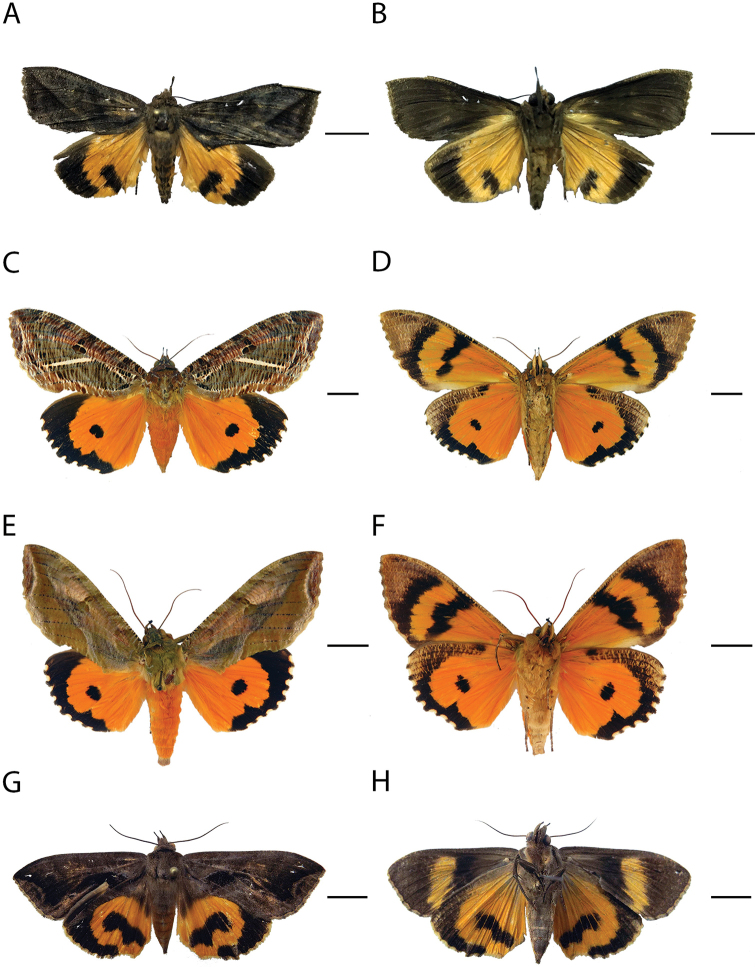
Species of *Eudocima* fruit-piercing moths in Colombia. **A, B** Dorsal and ventral view of *E. anguina* male **C, D** same for *E. apta* female **E, F** same for *E. apta* male **G, H** same for *E. collusoria* female. Scale bars: 1 cm.

#### Eudocima apta

Taxon classificationAnimaliaLepidopteraNoctuidae

(Walker, [1858]) (Ophideres)

94479117-7641-5807-859B-2C101B35A6A7

Fig. 2C–F

##### Material examined.

Colombia. **Antioquia**: 1♀; Medellín; 6.2518, -75.5636; Oct. 1993; N. Monsalve leg.; MFLG 46650. 1♂; same locality; Aug. 1967; R. Velez leg.; MFLG 46651. 1♂; Yarumal; vda. Corcovado, Alto de Ventanas; 7.0743, -75.4436; 2020 m; 22–26 Jun. 2015; ICN. **Boyacá**: 1♀; Cerinza; Parque principal; 5.9632, -72.9626; 2750 m; 31 Oct. 2014; E. Corredor leg.; UNAB. **Cauca**: 2♂♂, 1 ♀; Belalcazar [Páez]; PNN Nevado del Huila, Termales, Ins. Pol. Irlanda; 2.6547, -75.9928; 2800 m; 2 Sep. 1980; C. Bohórquez leg.; light trap; ICN-80 1783, 1783, 1784. **Cundinamarca**: 1♀; Anapoima; Andalucía; 4.5489, -74.5352; 700 m; 14 Sep. 2009; M. Galindo leg.; entomological net; UNAB. 1♂; same locality; 670 m; 1 Nov. 2008; D. Ramirez leg.; entomological net; UNAB. 1♀; Bogotá; Barrio Quinta Ramos; 4.5775, -74.0923; 2555 m; 5 Nov. 2015; J. Rincon leg.; hand collecting; UNAB. 1♂, 1♀; Bogotá; Chapinero; 4.6097, -74.0818; 1 Nov. 1963–1 Feb. 1964; S. Restrepo leg.; MPUJ_ENT 0019402, 0019395. 1♀; Bogotá; Las Villas; 4.6097, -74.0818; 2630 m; 1 Mar. 1985; O. Ricardo leg.; MPUJ_ENT 0019406. 1♂; same locality; 8 Mar. 1985; V. Leonardo leg.; MPUJ_ENT 0019409. 1♂, 1♀; same locality; 17 Mar. 1986–17 Jun. 1988; JFLC. 1♂, 3♀♀; same locality; NHMUK. 1♂; Bogotá; U. La Salle; 4.6097, -74.0818; 17 Jun. 1977; J. Restrepo leg.; MLS 4684. 1♀; Bogotá; Univ. Nal. Col.; 4.6333, -74.0833; 2562 m; 18 Nov. 2014; Hernández leg.; hand collecting; UNAB. 1♂; same locality; 21 Apr. 2016; V. Ramirez leg.; UNAB. 1♂; same locality; 1 Nov. 2012; F. Ariza leg.; hand collecting; UNAB. 1♂; same locality; 12 Oct. 2016; Jaramillo leg.; entomological net; UNAB. 1♂; same locality; 10 Nov. 2015; L. Lemus leg.; hand collecting; UNAB. 1♂; same locality; 8 Mar. 2012; C. Peña leg.; entomological net; UNAB. 1♀; same locality; 2 Sep. 2014; A. Gamba leg.; hand collecting; UNAB. 1♀; same locality; 10 May 2012; C. Pinilla leg.; hand collecting; UNAB. 1♀; same locality; 12 Nov. 2015; A. Arevalo leg.; hand collecting; UNAB. 1♀; Bogotá; Timiza; 4.6088, -74.1554; 2600 m; 8 Jul. 2015; P. Osorio leg.; CTNI 183. 1♂; Gachalá; vda. Tunja; 4.8924, -73.5066; 1500 m; 1–3 Sep. 2015; ICN. 1♂; Mosquera; 4.7059, -74.2302; 28 Aug. 1979; N. Ruiz leg.; light trap; CTNI 222. 1♀; San Antonio del Tequendama, Santandercito; 4.6, -74.35; 1 May 1960; MPUJ_ENT 0019397. 1♂; Silvania; 4.4538, -74.3642; 140 m; 19 Nov. 2015; C. Hernández leg.; light trap; UNAB. 2♂♂, 1♀; Soacha, Km. 8, vía-Mosquera; RN Chicaque, Quebrada el Carmen; 4.5921, -74.2763; 2 Ago. 2016.; D. Cualla leg.; MPUJ_ENT 0048742, 0048765, 0048766. **La Guajira**: 1♂; 11.544, -72.9072; ICN. **Norte de Santander**: 2♀♀; Santo Domingo de Silos; Páramo de Berlin; 7.2378, -72.8103; 3171 m; 2 Jul. 2016; J. Montes leg.; hand collecting; MFLG. 1♂; same data; CTNI. **Santander**: 1♀; Floridablanca; 7.0622, -73.0864; 1 Sep. 1980; W. Olarte leg.; MHN-UIS. 1♂, 2♀♀; Rionegro; vda. La Paz, Finca La Esperanza; 7.3247, -73.1751; 1105 m; 5 Jul. 2016; J. Montes leg.; on *Citrus* sp. crop, hand collecting; CTNI. **Tolima**: 1♀; Armero; Hacienda El Dormilón; 4.9887, -74.8813; 180 m; 1 Oct. 2000; G. Fagua leg.; Malaise trap; MPUJ_ENT 0045647. 1♀; Melgar; vda. Aguila Media, Finca Santa Lucia; 4.1667, -74.5667; 1163 m; 4 Mar. 2012; J. Restrepo leg.; hand collecting; UNAB. **Valle del Cauca**: 1♀; Anchicayá; 3.6186, -76.9133; 400 m; 28 Aug. 1967; MUSENUV 14837. 1♀; same locality; 16 Jul. 1977; MUSENUV 14836. Without specific locality: 1♂; MLS 8386. 1♂; MPUJ_ENT 0019412.

##### Comments.

This species is easily distinguished from the other neotropical members of the genus by its circular black discal spot on the hindwings. Janzen and Hallwachs (2009) recorded larvae of *E. apta* as feeding on *Disciphania heterophylla* Barneby and *Cissampelos pareira* L. (Menispermaceae), whereas Van Bael et al. (2004) recorded in Panama *Odontocarya tamoides* Miers (misspelled as *O. lamnoides*), also Menispermaceae, as a host plant. In Colombia, *C. pareira* has a wide distribution, however *O. tamoides* is restricted to the lowlands of the Caribbean Plain, the Pacific region and the Magdalena Valley (Bernal et al. 2019). Adults of *E. apta* have been reported to affect several crops: *Citrus sinensis* [L.] Osbeck (Rutaceae) and genus *Vitis* (Vitaceae) in Cuba and Dominican Republic (Robinson et al. 2010); *Carica papaya* L. (Caricaceae) in Mexico (Hernández-Ruiz et al. 2017). Recently, it was recorded from citrus crops in Colombia (Montes et al. 2018). It has been collected using light traps (Brou 1994, Janzen and Hallwachs 2009).

##### Distribution.

Widespread in the New World, from southern United States and the Caribbean to Brazil, the South Atlantic Islands and north of Chile (Angulo and Jana-Sáenz 1983; Brou 1994, Zilli and Hogenes 2002, Brou and Núñez 2013). Powell and Brown (1990) recorded *E. apta* up to an elevation of 3900 m. In Colombia, it has been recorded in several localities within the eastern cordillera and eastern slope of the central cordillera in a wide elevational range.

##### Remarks.

Traditionally it has been incorrectly identified as *Eudocima materna* (Linnaeus, 1767) (e.g., Costa Lima 1950: fig. 158). However, *E. materna* is distributed in the Old World. Zilli and Hogenes (2002) provided a rationale for considering *E. apta* as a valid species and not a synonym of *E. materna*.

#### Eudocima collusoria

Taxon classificationAnimaliaLepidopteraNoctuidae

(Cramer, 1777) (Phalaena (Noctua))

EE195C43-041F-51EB-A861-BB158C5835EC

Fig. 2G, H

##### Material examined.

Colombia. **Cundinamarca**: 1♀; Silvania; Km. 31 Bogotá-Silvania; 4.4212, -74.3888; 1386 m; 8 May 2016; K. Medina leg.; entomological net; UNAB.

##### Comments.

The “m”-shaped band in the hindwings resembles those of *E. memorans* and *E. colubra*, but it differs in having the inner margin straighter. The forewings do not have pale transverse bands as in *E. memorans*. Lalanne-Cassou and Silvain (2003) report this species in primary forest of French Guiana. The hostplant and life cycle are unknown. See also comments under *E. anguina*.

##### Distribution.

Neotropical (Zilli and Hogenes 2002): Surinam (Cramer 1777), French Guiana (Lalanne-Cassou and Silvain 2003) and Colombia.

##### Remarks.

One male specimen was examined from Cundinamarca. It is recorded for the first time from Colombia.

#### Eudocima colubra

Taxon classificationAnimaliaLepidopteraNoctuidae

(Schaus, 1911) (Trissophaes)

CFCAAB7D-3755-5233-A08E-8D035015A92F

Fig. 3A–C

##### Material examined.

Colombia. **Antioquia**: 1♀; Medellín; 6.2518, -75.5636; May 1984; F. Serna leg.; On a wall; MFLG 46641. 1♀; Yarumal; vda. Corcovado, Alto de Ventanas; 7.0743, -75.4436; 2020 m; 22–26 Jun. 2015; ICN. **Chocó**: 1♂; Río Tamaná, El Tigre; 5.15, -76.2166; 97 m; 09 Feb.; G.M. Palmer leg.; NHMUK.

##### Comments.

Marked sexual dimorphism in the coloration of forewings. The species has a sinuous band on the posterior wings in the form of an “m”, similar to those of *E. memorans* and *E. collusoria*. Janzen and Hallwachs (2009) record larvae of *E. colubra* as feeding on *Disciphania calocarpa* Standl. (Menispermaceae) in Costa Rica.

##### Distribution.

Costa Rica, Peru (Schaus 1911) and Colombia.

##### Remarks.

Three specimens were examined from the departments of Antioquia and Chocó. It is recorded from Colombia for the first time.

**Figure 3.**
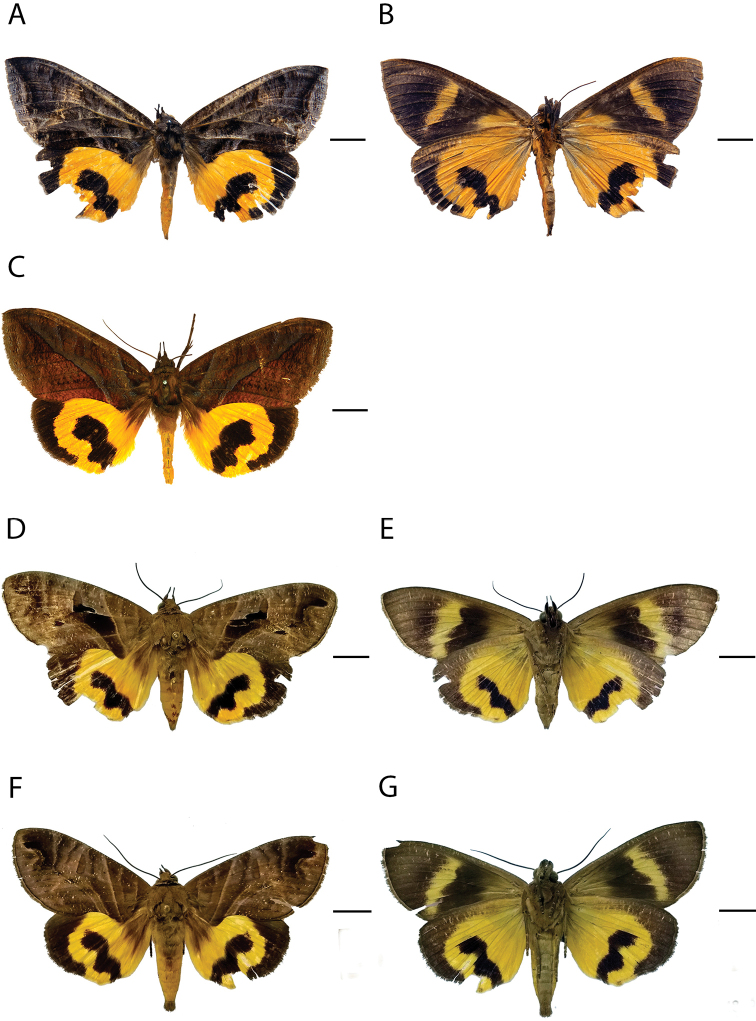
Species of *Eudocima* fruit-piercing moths in Colombia. **A, B** Dorsal and ventral view of *E. colubra* female **C** dorsal for *E. colubra* male **D, E** same for *E. memorans* female **F, G** same for *E. memorans* male. Scale bars: 1 cm.

#### Eudocima memorans

Taxon classificationAnimaliaLepidopteraNoctuidae

(Walker, [1858]) (Ophideres)

3EAE3F9B-2F2F-5FC6-BB72-09D46AA12DB2

Fig. 3D–G

##### Material examined.

Colombia. **Antioquia**: 1♂; Valle de Aburrá; 6.2833, -75.5; May 1953; F. Gallego leg.; MFLG 46653. **Bolivar**: 1♀; Cartagena; 10.3997, -75.5144; L. Cortes leg.; MHN-UIS. **Caldas**: 1♂; Chinchiná; 4.9728, -75.6819; Oct. 1955; F. Gallego leg.; On a road; MFLG 46652. **Cundinamarca**: 1♀; Bogotá; 4.6097, -74.0818; 1 Feb. 1962; MPUJ_ENT 0019396. 2♂♂, 1♀; Pacho; Plaza de Toros; 5.1361, -74.1602; 1740; 7 Jan. 1992, 26 Mar. 1992; G. Patrick leg.; HNSA. **Valle del Cauca**: 1♂, 1♀; Anchicayá; 3.6186, -76.9133; 400 m; 17 Jun. 1977–10 Sep. 1977; Dahners leg.; MUSENUV 14841, 14849. 1♀; R[io] Dagua; 3.8455, -77.0609; W. Rosenberg leg.; NHMUK. 1♂, 1♀; [Valle del] Cauca, Juntas [Río Dagua]; 3.6164, -76.73222; 1897–1898; M. de Mathan leg.; NHMUK.

##### Comments.

The hindwings show an “m”-shaped band with a distinctly sinuous inner margin. Unlike *E. serpentifera*, this band reaches the wing margin. Forewing with oblique pale bands. Hostplant and life cycle unknown.

##### Distribution.

Neotropical (Zilli and Hogenes 2002). Described from “the western coast of the Americas” (Walker, [1858]), Brou (2006) suggested that the original specimens probably originated from Ecuador.

##### Remarks.

This species is recorded for the first time from Colombia.

#### Eudocima procus

Taxon classificationAnimaliaLepidopteraNoctuidae

(Cramer, 1777) (Phalaena (Noctua))

6E804420-ECA4-56CC-89DB-784DC17AAB95

Fig. 4A–D

Ophideres columbina Guenée, 1852
Ophideres scabellum Guenée, 1852
Acacallis procax ; Hübner, [1823] [misspelling]

##### Material examined.

Colombia. **Antioquia**: 1♂; Medellín; 6.2518, -75.5636; Apr. 1989; F. Cuartas leg.; entomological net; CEUA. 1♀; same locality; 26 Apr. 1969; F. Mosquera leg.; CTNI 99. 1♂; Medellín; 6.2518, -75.5636; 28 Jun. 1938; J. Gates-Clarke leg.; MFLG. 1♂; same locality; Sep. 1958; F. Gallego leg.; entomological net; MFLG 46647. 1♂; same locality; Oct. 1976; R. Velez leg.; on a door; MFLG 46646. 1♂; same locality; May 1946; F. Gallego leg.; On a wall; MFLG 46645. 1♂; same locality; 1538 m; 28 Mar. 1998; S. Blandon leg.; On a wall; MFLG 46649. 1♂; Sabaneta; Barrio La Doctora; 6.15, -75.5833; 1570 m; May 2005; entomological net; CEUA. 2♀♀; Santa Fe de Antioquia; 6.5569, -75.8281; Nov. 1981; M. Monzón leg.; On Papaya; MFLG 46642, 46643. 1♂; Valle de Aburrá; 6.2833, -75.5; Oct. 1943; F. Gallego leg.; MFLG. 1♂; Valle de Aburrá; 6.2833, -75.5; Feg. 1946; F. Gallego leg.; undergrowth; MFLG 46648. 2♀♀; Yarumal; vda. Corcovado, Alto de Ventanas; 7.0743, -75.4436; 2020 m; 22–26 Jun. 2015; ICN. **Boyacá**: 3♂♂; Arcabuco; vda. Peñas Blancas; 5.723, -73.4678; 2674 m; 17–19 Sep. 2017; ICN. 1♂; Sotaquirá; 5.7618, -73.2859; 9 Aug. 1969; J. Alba leg.; UNAB. 1♂; Turmequé; Villa Nely; 5.3062, -73.5088; 2800 m; 27 Apr. 2003; S. Angel leg.; entomological net; UNAB. **Caldas**: 1♀; Florida; 4.9931, -75.7439; Sep. 1963; F. Gallego leg.; On a wall; MFLG 46644. **Cauca**: 1♂; Guapi; PNN. Gorgona-Poblado; 2.9683, -78.1844; 10 m; 19–22 Oct. 2010; H. Calero leg.; MUSENUV B16. **Cundinamarca**: 1♂; Agua de Dios; 4.3584, -74.69; 1 Jun. 1997; J. Gutierrez leg.; UNAB. 1♀; Beltrán; vda. La Esperanza; 4.8, -74.75; 250 m; 28 Apr. 2012; H. Rojas leg.; hand collecting; UNAB. 1♂; Bogotá; 4.6097, -74.0818; 17 May 1985; J. Cañon leg.; MLS 6677. 1♀; same locality; 18 Jun. 1905; R. Becerra leg.; MLS 8507. 1♂; same locality; 2 Oct. 1970; W. Prieto leg.; MLS 4683. 1♂; same locality; 17 Jul. 1961; J. Berecibar leg.; MPUJ_ENT 0045656. 4♂♂, 4 ♀♀; Bogotá; Chapinero; 4.6097, -74.0818; 30 Apr. 1962–12 Jun. 1962; E. Carvajalino leg.; MPUJ_ENT 0019404, 0019393, 0019408, 0019403, 0019401, 0045655, 0045657, 0045658. 1♂, 1♀; same locality; 14 -17 Apr. 2016; D. Cualla leg.; MPUJ_ENT 0045648, 0045652. 1♂; same locality; Oct. 1972; MPUJ_ENT 0045653. 1♀; same locality; 4.6097, -74.0818; 25 Jul. 2004; D. Corredor leg.; MPUJ_ENT 0019400. 1♂; same locality; 26 Jul. 1949; B. Diez leg.; MPUJ_ENT 0019405. 1♂; same locality; 13 Jun 1965; Amézquita leg.; CTNI 99. 1♀; same locality; 19 Apr. 1977; I. Zenner leg.; CTNI 99. 1♀; same locality; Oct. 1946; CTNI 99. 1♂; same locality; 25 Mar. 1947; CTNI 99. 1♂; same locality; 1 Oct. 1945; CTNI 100. 1♀; same locality; 11 Jun 1986; JFLC. 1♂; same locality; 1 Jul. 1996; J. F. Le Crom leg.; JFLC. 1♀; same locality; Birchall leg.; NHMUK. 1♀; same locality; 1918.; M. Apolinar leg.; NHMUK. 22♂♂, 5♀♀; same locality; NHMUK. 1♀; same locality; 1 Jul. 1967; UNAB. 1♂; same locality; Jul. 1969; UNAB. 1♂; same locality; 8 May 1934; H. Pinzon leg.; UNAB. 1♂; same locality; 20 Nov. 1981; C. Orjuela and E. Mejia legs.; UNAB. 1♂; same locality; 9 May 1993; A. Diego leg.; UNAB. 1♀; same locality; 15 Apr. 1994; A. Tovar leg.; UNAB. 1♀; same locality; 22 Apr. 1984; C. Torres and F. Bernal legs.; UNAB. 1♂; same locality; May 1998; O. Castellanos leg.; UNAB. 1♂; same locality; 6 Mar. 1972; E. Gonzalez leg.; UNAB. 1♀; same locality; 20 Sep. 1975; E. Vargas leg.; UNAB. 1♀; same locality; 10 Apr. 1998; M. Arcos leg.; UNAB. 1♀; same locality; 20 Apr. 1975; A. Alarcon leg.; UNAB. 1♂; same locality; 9 Apr. 1995; L. Palacios leg.; UNAB. 1♂; same locality; 23 Apr. 1975; P. Acevedo leg.; UNAB. 1♂; Bogotá; Engativá; 4.7011, -74.1132; A. Casas leg.; UNAB. 1♂; Bogotá; Univ. Nal. Col.; 4.6333, -74.0833; 2600 m; 1 Feb. 2010; R. Forero leg.; hand collecting; UNAB. 1♂, 1♀; same locality; 8–10 Mar. 2014; A. Gamboa and J. Velásquez legs.; UNAB. 1♂; Cajicá; 4.9128, -74.0526; 17 Jun. 1975; A. Acosta leg.; hand collecting; UNAB. 1♂; same locality; 19 Sep. 1981; J. Rojas leg.; UNAB. 1♂; Fusagasugá; 4.3365, -74.3638; 27 Mar. 1972; L. Espinosa leg.; UNAB. 2♂♂; Gachalá; vda. Tunja; 4.8924, -73.5066; 1500 m; 1–3 Sep. 2015; ICN. 1♂; Girardot; 4.318, -74.835; 23 Nov. 1994.; S. Pulgarin leg.; UNAB. 1♂; Guaduas; Puerto Bogotá, Finca Altavista; 5.0743, -74.5985; MPUJ_ENT 0019410. 1♂; La Palma; 5.3173, -74.43; 14 Jun. 1978; I. Zenner leg.; On *Pinus* sp.; CTNI 99. 1♂; La Vega; 4.9738, -74.3448; 27 May 1969; A. Perez leg.; UNAB. 1♀; same locality; 1969; A. Perez leg.; UNAB. 1♂; same locality; 21. Mar. 1994.; H. Ramirez leg.; UNAB. 2♂♂; Medina; 4.5, -73.3333; 500 m; A. H. Fassl leg.; NHNUK. 1♀; Mosquera; 4.7059, -74.2302; 20 Apr. 1979; I. Zenner leg.; light trap; CTNI 99. 6♂♂, 12♀♀; Pacho; Plaza de Toros; 5.1361, -74.1602; 1740; 12 Jan.-5 Apr. 1992; G. Patrick leg.; HNSA. 1♂; Pandi; 4.1803, -74.471; 12 Nov. 1995; T. Corredor leg.; UNAB. 1♂; Sibaté; 4.4491, -74.2829; 10 Feb. 1990; I. Posada leg.; UNAB. 2♂♂; Soacha; 4.5794, -74.2168; 19 Mar. 1994, 21 Apr. 1969; UNAB. 2♂♂, 2 ♀♀; Soacha, Km. 8, vía-Mosquera; RN Chicaque, Quebrada el Carmen; 4.5921, -74.2763; 1 Ago. 2016.; D. Cualla leg.; MPUJ_ENT 0048768–0048771. 1♂; Tabio; 4.9351, -74.1021; Mar. 1986; D. Avellaneda leg.; UNAB. 1♂; Tibacuy; Cerro Quinini; 4.3058, -74.5164; Jun 1998; J. F. Le Crom leg.; JFLC. 1♀; Tocaima; 4.4607, -74.6572; 6 Nov. 1993; A. Guerrero leg.; UNAB. 1♂; Villeta; 5.0001, -74.505; 28 Jun 1965; E. Olivos leg.; CTNI 99. 1♀; same locality; 5 Jan. 1982; Bohórquez leg.; UNAB. **La Guajira**: 1♀; 11.544, -72.9072; ICN. **Meta**: 1♂; Villavicencio; 4.142, -73.6266; M. Apolinar leg.; NHMUK. 1♂; same locality; 10 Dic. 1981; G. Rodríguez leg.; UNAB. 1♀; same locality; 4.06, -73.4522; 5 Jun. 2015; O. Vargas leg.; UNAB. 1♂; Ober Río Negro; 4.2602, -73.8105; 800 m; A. H. Fassl leg.; NHMUK. **Norte de Santander**: 2♂♂; Santo Domingo de Silos; Páramo de Berlin; 7.2378, -72.8103; 3171 m; 2 Jul. 2016; J. Montes leg.; hand collecting; MFLG. 1♀; same data; CTNI. **Putumayo**: 2♂♂, 3♀♀; Mocoa; 1.1528, -76.6521; 26 Feb. 1972; MPUJ_ENT 0019398, 0045649–0045651, 0045654. 1♂, 1 ♀; Orito; 0.6675, -76.873; 26 Feb. 1972; MPUJ_ENT 0045645, 0019399. **Quindío**: 1♀; Río “Nauarco” [Río Navarco]; 4.62, -75.5881; NHMUK. **Santander**: 1♂; Bucaramanga; 7.1253, -73.1197; 1 Aug. 1979; W. Olarte leg.; MHN-UIS. 1♂; same locality; 1 Aug. 1978; W. Olarte leg.; MHN-UIS. 1♀; same locality; 15 May 1998; M. Estupiñan leg.; MHN-UIS. 2♂♂, 1 ♀; Rionegro; vda. La Paz. Finca La Esperanza; 7.3247, -73.1751; 1105 m; 5 Jul. 2016; J. Montes leg.; on *Citrus* sp. crop, hand collecting; CTNI. 1♀; Vélez; 6.2327, -73.7258; Jul. 1998; E. Espitia leg.; CTNI 99. **Tolima**: 1♂; Chaparral; 3.75, -75.5833; 3 Jul. 1969; J. Bedoya and H. Ruiz legs.; UNAB. 1♂; Espinal; 4.1492, -74.8843; 10 May 1969; Rojas leg.; UNAB. 1♀; Falan; 5.0795, -74.957; 18 Mar. 1990; O. Ferrer leg.; UNAB. 1♂; Melgar; 4.2048, -74.6408; 22 Sep. 1970; Salazar leg.; MLS 4677. 1♂; same locality; 3 May 1969; C. Forero leg.; UNAB. 1♀; same locality; 2 May 1993; S. Avendaño leg.; UNAB. **Valle del Cauca**: 3♂♂, 2♀♀; Anchicayá; 3.6186, -76.9133; 1000 m; 12 May 1975–28 Aug. 1976; MUSENUV 14831–14835. 1♂; Buga; Perímetro urbano; 3.9008, -76.2978; Nov. 1977; R. Torres leg.; MUSENUV 14842. 1♂; Cali; 3.4372, -76.5225; 1000 m; 15 Jun. 1975; MUSENUV 14843. 4♀♀; [Valle del] Cauca, Juntas [Río Dagua]; 3.6164, -76.73222; 1897–1898; M. de Mathan leg.; NHMUK. Without specific locality: 2♂♂, 4♀♀; CTNI 99, MLS 1968, 6674, MPUJ_ENT 0019442, 0019443, 0045646.

##### Comments.

It is easily distinguished from the other species of *Eudocima* by having two sinuous bands on the hindwing, in addition to a black basal band, which confer a somewhat checkered appearance. Caballero et al. (1994, as *O. scabellum*) recorded larvae of *E. procus* from *Odontocarya tamoides* (= *O. paupera*) (Menispermaceae) in Honduras. Adults were recently observed in Colombia in citrus orchards (Montes et al. 2018).

##### Distribution.

Widely distributed, with records from Central America to southern Brazil (Guenée 1852, Druce 1881–1900, Zilli and Hogenes 2002, Zaspel and Branham 2008). In the present work, specimens from several localities, mainly of the eastern and central cordilleras, were found. Widespread in Colombia.

##### Remarks.

Gallego (1946) reported this species (as *Othereis procus*, genus misspelled) to be frequently found in buildings of Medellín during the first half of the twentieth century. This species seems to be adapted to urban ecosystems as it is frequently attracted to city lights or even to boats near the Brazilian coast (Alves et al. 2019).

**Figure 4.**
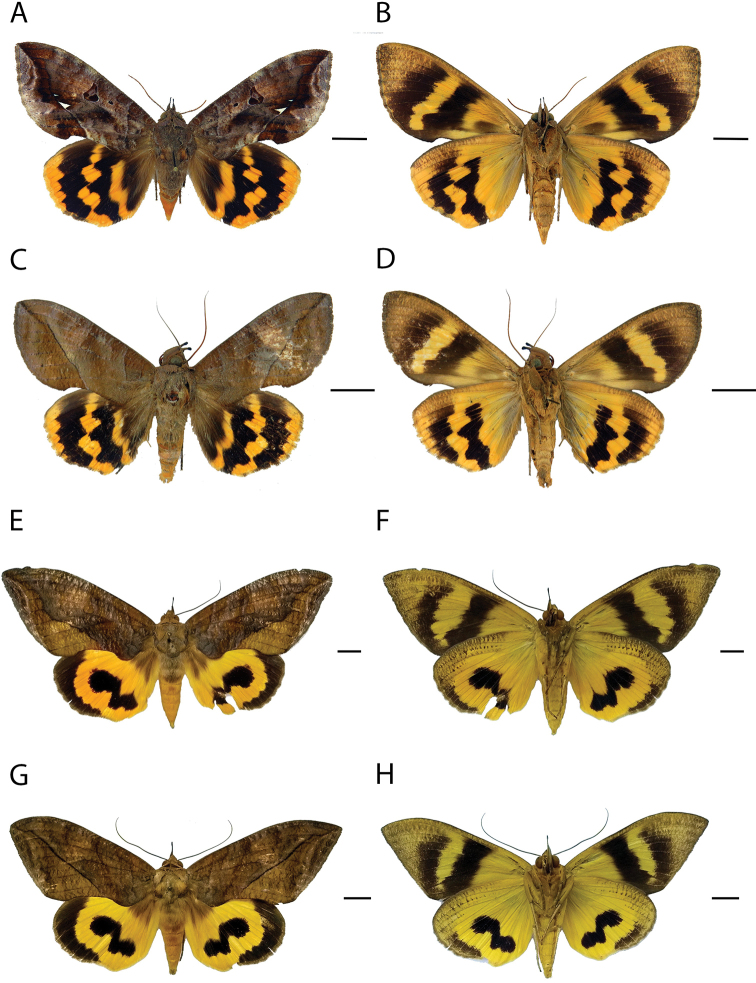
Species of fruit-piercing moths *Eudocima* in Colombia. **A, B** Dorsal and ventral view of *E. procus* female **C, D** same for *E. procus* male **E, F** Dorsal and ventral view of *E. serpentifera* female **G, H** same for *E. serpentifera* male. Scale bar: 1 cm.

#### Eudocima serpentifera

Taxon classificationAnimaliaLepidopteraNoctuidae

(Walker, [1858]) (Ophideres)

842FAE1E-BDA7-5A96-B39B-6FCCBC8FF6EB

Fig. 4E–H

Ophideres raphael Dugès, 1896

##### Material examined.

Colombia. **Antioquia**: 1♂, 1 ♀; Valle de Aburrá; 6.2833, -75.5; Sep. 1945-Sep. 1952; F. Gallego leg.; MFLG 46654, 46656. **Cauca**: 1♂; Guapi; PNN. Gorgona-Playa Blanca; 2.9484, -78.1842; 52 m; 19–22 Oct. 2010; H. Calero leg.; Van Someren-Rydon Trap; MUSENUV. **Cundinamarca**: 1♀; Bogotá; U. La Salle; 4.6097, -74.0818; 3 Dic. 1973; M. Nicéforo leg.; MLS 4672. 1♂; Pacho; Plaza de Toros; 5.1361, -74.1602; 1740; 7 Mar. 1992; G. Patrick leg.; HNSA. **Putumayo**: 1♂; Orito; 0.6675, -76.873; 26 Feb. 1972; MPUJ_ENT 0019407. **Tolima**: 1♂; Libano; 4.9217, -75.0622; Jul. 1956; F. Gallego leg.; MFLG 46655. **Valle del Cauca**: 2♂♂, 1 ♀; Anchicayá; 3.6186, -76.9133; 28 Nov. 1975- 20 Nov. 1976; MUSENUV 14846–14848.

##### Comments.

This species has a sinuous “m”-shaped band on the hindwings. Unlike other species with similar pattern on the hindwings such as *E. memorans*, *E. collusoria* and *E. colubra*, in *E. serpentifera* the “m”-shaped band does not reach the wing margin. Janzen and Hallwachs (2009) reported *D. calocarpa* (Menispermaceae) as its hostplant. Adults have been found feeding on *C. papaya* (Caricaceae) and *Citrus* in Mexico (Robinson et al. 2010; Hernández-Ruiz et al. 2017). In Mexico, adults are active from April to November and are commonly collected with light traps (Chamé-Vásquez and Jimenez 2009).

##### Distribution.

Widely distributed in Tropical America. Walker ([1858]) describes this species from the Dominican Republic and Brazil. Additionally, there are occasional records of this species from the southern United States (Brou 2006). In Mexico, it occurs in an elevational range between 150 and 3000 m (Chamé-Vásquez and Jimenez 2009).

##### Remarks.

This species is recorded for the first time from Colombia.

## Discussion

### Spatial and temporal distribution

The collections examined essentially consist of holdings from the Andean region. It is no surprise then that 94% of records are from the Andes, mainly the eastern mountain chain, with 65%, and 35% solely from Bogotá city. The Caribbean and Pacific regions have only three records each, and most of the Amazon region and Orinoquia are not represented in the sample; *Eudocima* moths are known only from three locations in the Amazonian foothills in the departments of Putumayo and Meta.

**Figure 5.**
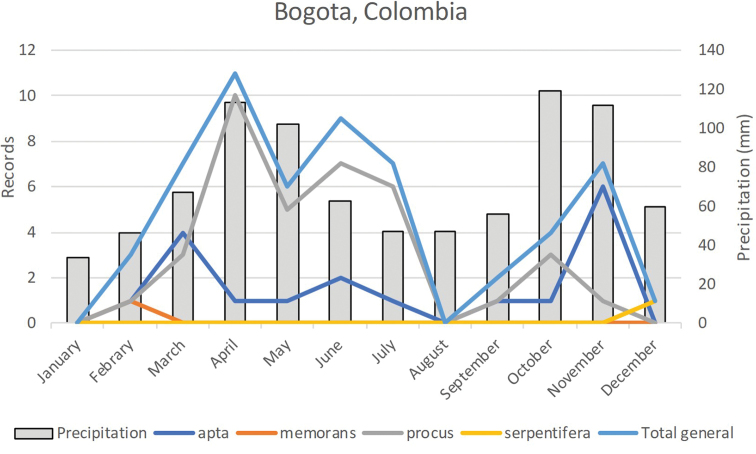
Records of *Eudocima* moths in the city of Bogotá, annual distribution vs precipitation.

Based on collection data from the city of Bogotá, the most common location represented in our sample, it is evident that seasonality of these moths is mainly determined by precipitation. Captures appear to be low in December and January, which are the months of lowest rainfall, and sharply increase during March, when the rainy season begins. Both the annual distribution of precipitation and that of moths show a bimodal pattern (Fig. 5). The relationship between moths and precipitation has frequently been reported (e.g., Bhumannavar and Viraktamath 2012), since with the onset of rainfall the sprouting of host plants increases, and oviposition of hundreds of eggs per female is triggered (Cochereau 1977, Magar et al. 2015).

The known geographical distribution of species of *Eudocima* is considerably expanded with our data. For instance, *E. anguina* was only known from Costa Rica, making the present record the first of this species in South America; *E. collusoria* was only known from Suriname and French Guiana; the record of *E. colubra* was predictable as this was known previously from Costa Rica and Peru; and *E. memorans*, described from the western coast of the Americas, was also found in the eastern mountain chain of Colombia and the Caribbean coast.

The wide distribution of *Eudocima* species is related to both their strong flight capacity (Bhumannavar and Viraktamath 2012) and close relationship with plants of the family Menispermaceae (Fay 1996). The larvae of *Eudocima apta* feed on *Cissampelos pareira* and those of *E. procus* have been recorded from *Odontocarya tamoides*. Both plants are widely distributed latitudinally and at elevations from 0 to 2800 m in the Andean, Pacific and Caribbean regions (Parr et al. 2014), overlapping with the distribution of these moths. On the other hand, the known host plant of *E. serpentifera* and *E. colubra*, namely *Disciphania calocarpa*, is mainly found in Central America and has only been recorded from Colombia in the humid montane forest of Dagua, Valle del Cauca, on the Pacific coast (Parr et al. 2014).

### Perspectives

Research on *Eudocima* moths is intrinsically twofold and may develop along both conservationist and agricultural lines. The larvae of these moths in the Neotropics feed exclusively on wild lianas of the family Menispermaceae (Janzen and Hallwachs 2009). It is expected therefore that breeding populations of these moths are restricted to natural or semi-natural areas with sufficient extent of forest patches, which exposes them to high vulnerability due to the ongoing deforestation. Some species, such as *E. anguina*, *E. collusoria*, *E. colubra* and *E. memorans*, may even be facing a higher risk due to their trophic relationships with just one of few host plants. In fact, these species are already rare in collections.

On the other hand, *Eudocima* moths were recently recorded for the first time as occasional fruit pests in Latin America; *E. apta* and *E. serpentifera* on papaya in Mexico (Hernández-Ruiz et al. 2017) and *E. apta* and *E. procus* on citrus in Colombia (Montes et al. 2018). At least in Colombia, damage by these moths was previously unknown by farmers, so many questions now arise about their origin and frequency.

Records of occasional outbreaks of fruit-piercing moths affecting orchards such as those in Colombia and Mexico had already been reported by Cochereau (1977) for New Caledonia in Oceania. Cochereau (1977) observed and monitored changes in *Eudocima phalonia* (Linnaeus, 1763) populations for three years since 1968–1970 and recorded in 1969 that its population increased rapidly with the onset of rains, after a period of drought of several months, and caused damage of more than 90% in citrus production, while the normal rate was around 4% (Cochereau 1973, 1977). The drought event was prompted by the El Niño-Southern Oscillation (ENSO) episode of 1968–1969, which reduced rainfall on the island of New Caledonia (Benoit and Delcroix 2000).

Unusually dry periods such as those recorded by Cochereau (1977) are also known to occur during ENSO events in the Andean region, especially in the eastern mountain range and the Caribbean region of Colombia (Montealegre 2007), where they have the potential to boost populations of fruit-piercing moths. The outbreaks of these moths on citrus orchards recorded from several municipalities in Colombia (Montes et al. 2018) were most likely triggered by the 2014–2016 ENSO event. Van Bael et al. (2004) also reported an outbreak of *E. apta* in Panama on June 1998, which was apparently influenced by the ENSO episode of 1997–1998. In addition, outbreaks of *E. serpentifera* have been reported in Honduras during the rainy seasons in 2012 to 2014 and 2016 (Van Dort 2019). Outbreaks of other Lepidoptera taxa in the rainy season following ENSO events have also been recorded in Panama (Van Bael et al. 2004, Srygley et al. 2010, 2014).

During such unusually dry periods several factors may act together and affect the natural control of moths, increasing their populations. In fact, the emergence of parasitoids is known to decrease with increasing temperature and drought (Romo and Tylianakis 2013), and also the rate of parasitoidism was shown to decrease with greater variability in rainfall between years (Stireman et al. 2005). This is likely an outcome of the uncoupling between cycles of hosts and parasitoids, which favors moth outbreaks. Another factor is the unusual sprouting of some plant species after an ENSO event. With the onset of rainfall, the young plant tissue is also of better quality for herbivores, containing a greater amount of leaf nitrogen and lower concentration of secondary defensive compounds such as tannins and phenols (Shure et al. 1998). This allows the development of a greater number of larvae; therefore, the longer the dry season the more luxuriant the vegetation will be, to the advantage of moth populations (Srygley et al. 2010, 2014).

Preliminary evidence therefore suggests a relationship between rainfall following ENSO-related drought and demographic increase of moth populations. That being the case, outbreaks of moth pests such as fruit-piercing *Eudocima* in orchards are expected to become commoner in future reflecting the increased frequency of ENSO events associated with climate change (Timmermann et al. 1999).

**Figure 6.**
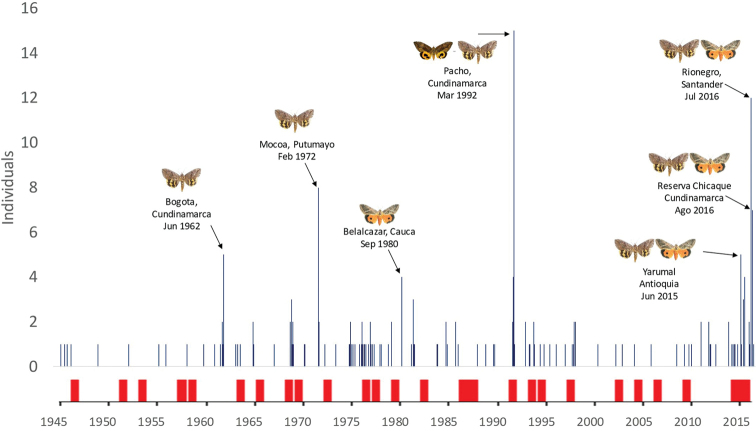
*Eudocima* records found in biological collections in Colombia. Vertical red lines indicate the duration of El Niño-Southern Oscillation ENSO events.

Although moth collections do not necessarily match exactly the distribution and abundance of species in the field, in the absence of strongly biasing factors (e.g., a ‘maniac’ collector selectively searching for particular species with exaggerate sampling effort) there is nonetheless an association between the commonness of a species in the field and the number of relevant vouchers deposited in collections. Accordingly, when several specimens of a species with the same locality and date are found, we expect such collection record to somewhat mark a natural population increase. Notably, when our records, which mostly originate from ecological sampling programs, are plotted along a timeline the increase of records matches the end of an ENSO event in most cases (Fig. 6). Remarkably, the high intensity ENSO event between 2014–2016 seems to have markedly increased the number of individuals in collections and outbreaks such as those reported in citrus (Montes et al. 2018).

Collections data provide invaluable information but there are some issues that cannot exclusively be addressed with these. Standardized long-term monitoring and sampling at night at selected sites with light traps, will be necessary to assess population dynamics over more ENSO cycles and to test the association between moth demography and climatic oscillations in the Neotropics. Surveys of host plants of fruit-piercing moths in natural areas will also shed light on several aspects of their biology, such as their life cycle and natural enemies. The importance of this information to preserve *Eudocima* diversity, especially regarding species exclusive of natural habitats, and reducing the damage caused to fruit orchards by pest species is evident.

## Supplementary Material

XML Treatment for Eudocima anguina

XML Treatment for Eudocima apta

XML Treatment for Eudocima collusoria

XML Treatment for Eudocima colubra

XML Treatment for Eudocima memorans

XML Treatment for Eudocima procus

XML Treatment for Eudocima serpentifera
